# Antibiotics Used for COVID-19 In-Patients from an Infectious Disease Ward

**DOI:** 10.3390/antibiotics12010150

**Published:** 2023-01-11

**Authors:** Felicia Sturza, Ștefan-Decebal Guță, Gabriel-Adrian Popescu

**Affiliations:** 1 Faculty of Medicine, University of Medicine and Pharmacy “Carol Davila”, 050474 Bucharest, Romania; 2 Department of Infectious Diseases, National Institute for Infectious Diseases “Prof. Dr. Matei Bals”, 021105 Bucharest, Romania; 3 Department of Microbiology, Cantacuzino National Military Medical Institute for Research and Development, 050096 Bucharest, Romania

**Keywords:** antibiotics, COVID-19, bacterial co-infection

## Abstract

Background: although the prevalence of bacterial co-infections for COVID-19 patients is very low, most patients receive empirical antimicrobial therapy. Furthermore, broad spectrum antibiotics are preferred to narrow spectrum antibiotics. Methods: in order to estimate the excess of antibiotic prescriptions for patients with COVID-19, and to identify the factors that were correlated with the unjustified antibiotic usage, we conducted an observational (cohort) prospective study in patients hospitalized with COVID-19 at the National Institute for Infectious Diseases “Prof. Dr. Matei Bals”, Bucharest, on an infectious disease ward, from November 2021 to January 2022. To evaluate the prevalence of bacterial co-infection in these patients, all positive microbiology results and concomitant suspected or confirmed bacterial co-infections, as documented by the treating doctor, were recorded. The patients were grouped in two categories: patients who received antibiotics and those who did not receive antibiotics, justified or not. Results: from the 205 patients enrolled in the study, 83 (40.4%) received antibiotics prior to being admitted to the hospital. 84 patients (41.0%) received antibiotics during their hospitalization; however, only 32 patients (15.6%) had signs and symptoms suggestive of an infection, 19 (9.3%) presented pulmonary consolidation on the computed tomography (CT) scan, 20 (9.7%) patients had leukocytosis, 29 (14.1%) had an increased procalcitonin level and only 22 (10.7%) patients had positive microbiological tests. It was observed that patients treated with antibiotics were older [70 (54–76) vs. 65 (52.5–71.5), *p* = 0.023, r = 0.159], had a higher Charlson index [4 (2–5) vs. 2 (1–4), *p* = 0.007, r = 0.189], had a severe/critical COVID-19 disease more frequently [61 (72.6%) vs. 38 (31.4%), *p* < 0.001, df = 3, X^2^ = 39.563] and required more oxygen [3 (0–6) vs. 0 (0–2), *p* < 0.001, r = 0.328]. Conclusion: empirical antibiotic treatment recommendation should be reserved for COVID-19 patients that also had other clinical or paraclinical changes, which suggest a bacterial infection. Further research is needed to better identify patients with bacterial co-infection that should receive antibiotic treatment.

## 1. Introduction

At the end of 2019, a novel coronavirus was identified as a causal agent for a cluster of atypical pneumonia cases in Wuhan, China [1]. This virus spread rapidly, causing the COVID-19 pandemic, which caused more than 6.6 million deaths world-wide by the time this article was published [2]. Over time, humanity has faced multiple pandemics, including the influenza pandemic of 1918, which caused millions of fatalities [3]. Bacterial co-infections can increase the severity of viral respiratory infections and represent an important mortality cause. These are present in approximatively 18–30% of patients admitted for influenza in intensive care units [4,5]. The role of bacterial co-infections in COVID-19 cases remains uncertain. It is estimated that 4.4% of hospitalized patients with a SARS-CoV-2 infection had bacterial co-infections upon admission, most frequently with *Staphylococcus aureus*, *Streptococcus pneumoniae*, *Haemophilus influenzae* and, even less commonly, atypical bacteria (*Mycoplasma pneumoniae*, *Chlamydia pneumoniae*) [6]. However, prolonged hospitalization increases the risk of hospital-acquired infections. The most common pathogens are *Pseudomonas aeruginosa*, *Klebsiella* spp., *S. aureus* and *Acinetobacter baumannii* [7,8].

Despite the low number of bacterial co-infection cases associated with COVID-19, most patients diagnosed with a SARS-CoV-2 infection received empirical antibiotic treatment, as shown in recent studies [9]. Notwithstanding, during a period when viral infections dominated the infectious disease pathology, antibiotic usage should have been significantly diminished. While in the EU the total consumption of antibiotics was approximatively 17% lower in 2020 compared to 2019, in Romania, the usage was only 3% lower [10,11]. Not only did Romania miss the opportunity to decrease antibiotic consumption, but inappropriate antibiotic usage has also increased; broad spectrum antibiotics were preferred to the detriment of narrow spectrum ones: penicillins and tetracyclines are used less, while fluoroquinolones (antibiotics for which the European Commission issued a decision to restrict usage, considering their associated risks) were used twice as much compared to the European average. Furthermore, in Romania in 2020, we were facing an unwanted excess of certain antibiotics, including macrolides and carbapenems [10]. Azithromycin usage has been increasing in Romania in the past few years and was the most used out-patient antibiotic in 2020, being 3 times more than in 2019, its prescription being closely related with the SARS-CoV-2 infection. Another concerning phenomenon is represented by the increase of cases of Gram-negative, carbapenem-resistant bacterial infections in COVID-19 patients in intensive care units, partially related to a 32% increase of meropenem consumption in 2020 compared to 2019 [10].

As we understand more about COVID-19, it is essential for prescribing physicians to differentiate between patients at risk of developing a bacterial co-infection, and to prescribe the necessary antibiotics when needed by understanding and accounting for associated risk factors (age, immunosuppression, etc.), along with using laboratory and imaging (leukocytosis, elevated procalcitonin, positive blood cultures and radiological findings suggestive of bacterial infections) [12,13].

## 2. Results

### 2.1. Clinical Features

Two hundred five consecutive patients with SARS-CoV-2 infections from a clinical infectious disease ward were included in the study, between 1 November 2021–31 January 2022. The age median was 66 years [interquartile range (IQR) 53–73] and 108 patients (52.7%) were women. A total of 152 patients (74.1%) had at least one comorbidity, with a Charlson index median of 3 (IQR 1–5). The most frequent comorbidities were cardio-vascular diseases, diabetes mellitus, cancer, chronic kidney failure, etc. When evaluating the severity of the disease, 30 patients (14.6%) had mild disease, 76 patients (37.1%) had moderate disease, 81 patients (39.5%) had severe disease and 18 patients (8.8%) had critical disease. Regarding rapid initiation of antiviral therapy, 174 (84.9%) patients received remdesivir, started within 7 (IQR 5–10) days of symptoms onset, and it was administered for 5 days in 164 (95.9%) patients. In total, 125 (61.3%) received dexamethasone, initiated within 9 (IQR 7–11) days of symptoms onset, and it was administered for 9 days (IQR 7–11); eighteen (8.8%) received anakinra, 17 (8.3%) received baricitinib and 15 (7.3%) received tocilizumab. Twenty-seven (13.2%) patients received casirivimab and imdevimab when the Delta variant (B.1.617.2) of SARS-CoV-2 was the dominant strain in our country (Table 1). No patient presented post-antibiotic *Clostridioides difficile* diarrhea while hospitalized.

### 2.2. Evaluation of Predictors for Bacterial Co-Infection and Antibiotic Use

Out of the 205 patients included in the study, 84 (41.0%) received antibiotics prior to being admitted (most frequently as monotherapy) and 36 (42.8%) of those received azithromycin. During hospitalization, in the clinical infectious disease ward, 84 patients (41.0%) received antibiotics, and, for 61 (72.6%) patients, they were administered as monotherapy. In total, 37 (18.0%) patients were treated with antibiotics, justified by clinical or paraclinical findings of bacterial infection, 47 (22.9%) patients received antibiotics unjustified, 121 (59.0%) of them were neither prescribed antibiotics, nor did they have a suspected or confirmed infection and there were no patients (0.0%) without antibiotic treatment while having arguments for bacterial infection. Eighteen patients (8.8%) were transferred to the intensive care unit (ICU).

Out of all the patients who received antibiotics, a very low number actually had clinical or paraclinical findings warranting antibiotic administration; at admission, 32 patients (15.6%) had associated symptoms suggestive of an infection, unexplained by the SARS-CoV-2 infection, 19 patients (9.3%) presented pulmonary consolidation on the CT scan, 24 patients (11.7%) had leukocytosis and 44 patients (35.5%) had a procalcitonin level greater than 0.05 ng/mL at admission (procalcitonin levels were available for 124 patients). Only 22 patients (10.7%) had positive microbiological tests and 10 of them were bacterial colonization (Table 2). Fifteen patients (7.31%) had a positive urine culture from which *Escherichia coli* (*n* = 10), *Enterococcus faecalis* (*n* = 4), *Providencia stuartii* (*n* = 1) and *Pseudomonas aeruginosa* (*n* = 1) were isolated. From respiratory samples, 6 patients (2.92%) had positive cultures with *Staphylococcus aureus* (*n* = 2), *Haemophilus influenzae* (*n* = 1), *Moraxella catarrhalis* (*n* = 1), *Streptococcus* spp. (group G) (*n* = 1), *Klebsiella pneumoniae* (*n* = 1), *Klebsiella oxytoca* (*n* = 1), *Enterobacter cloacae* (*n* = 1), *Pseudomonas aeruginosa* (*n* = 1) and *Acinetobacter baumannii* (*n* = 1). From blood cultures, 3 (1.4%) samples were positive with *Staphylococcus aureus* (*n* = 2) and *Enterococcus faecalis* (*n* = 1).

**Figure 1:**
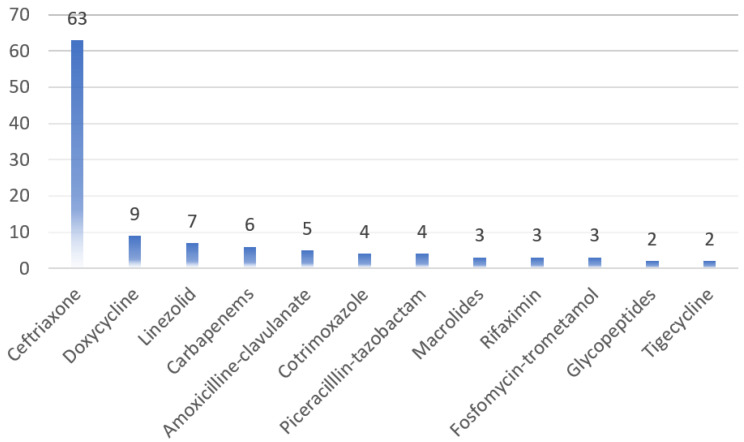
Antibiotic therapy in patients hospitalized with COVID-19.

On average, antibiotic treatment was initiated on day 10 (6–13) after the onset of the symptoms, while for 65 (77.3%) patients, antibiotic therapy was initiated 48 h after being admitted and was administered for 7 days (5–7). Most patients were treated with ceftriaxone (63/84, 75.0%). Other antibiotics used were doxycycline (9/84, 10.7%), carbapenems (6/84, 7.1%), linezolid (7/84, 8.3%), amoxicillin-clavulanate (5/84, 5.9%), piperacillin-tazobactam (4/84, 4.7%), cotrimoxazole (4/84, 4.7%), macrolides (3/84, 3.5%), rifaximin (3/84, 3.5%), Fosfomycin-trometamol (3/84, 3.5%), glycopeptides (2/84, 2.3%) and tigecycline (2/84, 2.3%) (Figure 1). Therefore, only 18 patients were prescribed antibiotics within the “access” group, while 84 patients received antibiotics within the “watch” group and 9 patients were treated with antibiotics within the “reserve” group (some patients were treated with more than one antibiotic).

**Figure 2:**
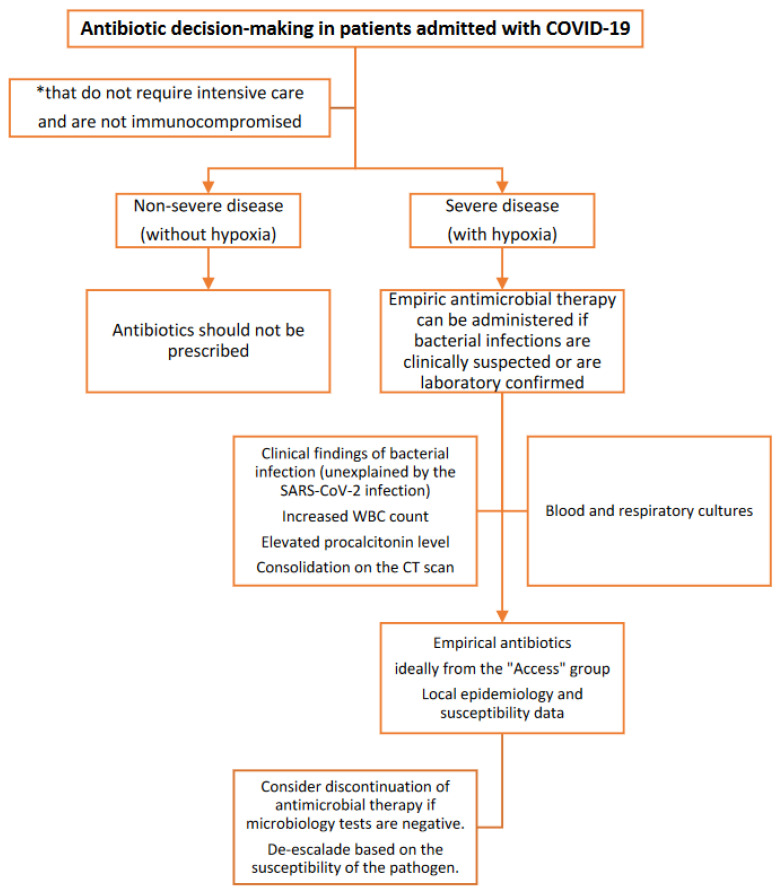
Antibiotic decision-making in patients admitted with COVID-19. * was a substitute for patients.

It was observed that patients treated with antibiotics were older [70 (54–76) vs. 65 (52.5–71.5), *p* = 0.023, r = 0.159], had a higher Charlson index [4 (2–5) vs. 2 (1–4), *p* = 0.007, r = 0.189], had a severe/critical COVID-19 disease more frequently [61 (72.6%) vs. 38 (31.4%), *p* < 0.001, df = 3, X^2^ = 39.563] and required more oxygen [3 (0–6) vs. 0 (0–2), *p* < 0.001, r = 0.328]. Furthermore, the patients which received antibiotic treatment had a higher white blood count [(absolute value × 10^9^/L) 6.2 (4.6–8.6) vs. 5.1 (4.4–6.7), *p* = 0.015, r = 0.170] and they had more frequent leukocytosis [16 (19.0%) vs. 8 (6.6%), *p* = 0.006, df = 1, X^2^ = 7.418]. They also had a higher value of the C-reactive protein [69.9 (22.7–119.5) vs. 18.1 (6.1–49.5), *p* < 0.001, r = 0.370], of ferritin [536 (201.5–936.6) vs. 197.9 (69.8–509.6), *p* < 0.001, r = 0.247] and of interleukin-6 [127.6 (58.1–463.4) vs. 40.7 (16.7–122.6), *p* < 0.001, r = 0.286]. More patients who had a higher level of procalcitonin were treated with antibiotics [31 (48.4%) vs. 13 (21.7%), *p* = 0.002, df = 1, X^2^ = 9.695]. Although the median of the procalcitonin was 0.05, similar between those treated without and with antibiotics, the upper quartile of those not treated with antibiotics was 0.05, compared to 0.15 in those treated with antibiotics (*p* = 0.001, r = 0.224). The patients who received antibiotic treatment had more frequent signs and symptoms suggestive of a bacterial infection, unexplained by the SARS-CoV-2 infection [32 (38.1%) vs. 0 (0.0%), *p* < 0.001, df = 1, X^2^ = 54.622] and consolidation on the CT examination [16 (19.3%) vs. 3 (2.5%), *p* < 0.001, df = 1, X^2^ = 16.279]. It should also be noted that the patients who received antibiotic treatment were treated more frequently with corticosteroids [69 (82.1%) vs. 57 (47.1%), *p* < 0.001, df = 1, X^2^ = 25.694] and immunomodulating treatment with tocilizumab [11 (13.1%) vs. 4 (3.3%), *p* = 0.008, df = 1, X^2^ = 7.006] or anakinra [13 (15.5%), vs. 5 (4.1%), *p* = 0.005, df = 1, X^2^ = 7.966]. Patients who received antibiotics were more frequently associated with a bacterial infection documented by positive respiratory tract cultures [6 (7.1%) vs. 1 (0.8%), *p* = 0.02, df = 1, phi = 0.171). Patients treated with antibiotics did not have a lower risk of being admitted to an ICU [11 (61.1%) vs. 7 (38.9%), *p* = 0.069, X^2^ = 3.308] (Figure 2).

Multivariate analysis showed that antibiotic prescription was associated with older age (*p* = 0.049), higher Charlson comorbidity index (*p* = 0.015), increased oxygen requirement (*p* < 0.001), increased level of white blood cells (*p* = 0.016) and higher C-reactive protein (*p* < 0.001). This time, antibiotic therapy was not associated with increased levels of ferritin and interleukin-6 most likely because they were collected from a limited number of patients.

## 3. Discussion

Among the patients who were treated with antibiotics for COVID-19 pneumonia, only a small number had positive microbiological tests. Although patients treated with antibiotics had a more severe form of pneumonia and a higher oxygen requirement, antibiotic treatment did not prevent deterioration, ICU admission or mortality. Clinical features, radiologic findings, along with an increased white blood cell count and an elevated procalcitonin, point to a bacterial co-infection. In a similar study from Singapore, authors found that only 12.0% of the patients were treated with antibiotics, and out of those, only 3.6% had documented (clinical or microbiological) bacterial infections [14].

A meta-analysis showed that bacterial co-infection was present in 3.5% of cases on admission and a 14.3% rate of bacterial infections during hospitalization. Nevertheless, 71% of patients were prescribed antibiotics [15]. A cohort study showed a 3.2% rate of bacterial co-infections on admission and 6.1% during hospitalization [16]. In a different cohort study, a respiratory bacterial co-infection rate of 11.2% was identified [17]. A systematic review described a bacterial co-infection rate of 8%; however, 72% of patients received empirical antibiotic treatment [18]. The same trends of prescribing excess antibiotics, disproportionately from the documented bacterial co-infection cases, was identified by a different cohort study, where 43.8% of patients received antibiotics, while only 19.1% had suggestive arguments for their administration [19]. Researchers found a rate of hospital-acquired infections of 7.1% among patients with COVID-19 in Wuhan. The most frequent hospital-acquired infection was pneumonia (32.3%), followed by bacteriemia (24.6%) and urinary tract infections. The mortality of in-patients with COVID-19 with a hospital-acquired infection was 15.4%, compared to 7.3% for patients without associated bacterial infections [20]. In a cohort study in Barcelona, authors found microbiologically confirmed infections in 7.2% of cases, 3.1% being community-acquired infections, most frequently caused by *S. pneumoniae* and *S. aureus*, and 4.7% being hospital-acquired infections, most frequently caused by *P. aeruginosa* and *E. coli* [21]. Most studies only reported bacterial co-infections that were confirmed through microbiological tests. Although, in our study, we also included bacterial co-infections diagnosed through clinical criteria or non-specific paraclinical tests, there was still an excess of prescribing antibiotics for patients without signs of an associated bacterial co-infection. Current guidelines recommend stopping the empirical antibiotic if microbiological tests performed before the start of the antibiotic administration do not reveal any sign of bacterial growth [22].

Prescribing physicians tend to treat elderly patients with multiple comorbidities and a severe form of pneumonia with antibiotics [23]; however, this proved ineffective because it did not decrease the necessity to admit patients into an ICU. Furthermore, antibiotics do not lack adverse effects. Overutilizing antibiotics during the COVID-19 pandemic will lead to an emergence of multi-drug resistant bacteria. Antimicrobial resistance has proven to be another global pandemic, with millions of infections with antibiotic resistant bacteria and over 1.27 million deaths annually [24,25]. Present estimates put the number of yearly deaths caused by resistant bacterial infections at around 10 million by 2050 [26]. Adherence to antimicrobial stewardship programs is declining as physicians are faced with critical patients, and the diagnosis of a bacterial superinfection is often hard to make [27]. Most of our patients have been treated with antibiotics from the “watch” group (i.e., ceftriaxone) from the WHO classification, not with first line antibiotics. WHO recommended that, by 2023, a minimum of 60% of total antibiotic consumption should be first line antibiotics (“access”), those with the lowest risk to induce the appearance of resistances [28]. In Romania, in 2020, first line antibiotics were prescribed in 49.9% of cases [10]. It is important to reevaluate the need to administer an antibiotic for SARS-CoV-2 infections daily and to deescalate antibiotic therapy.

White blood count, C-reactive protein and procalcitonin, having a specificity of over 82% and a low sensitivity under 40%, can predict the absence of a bacterial co-infection. A cohort study on patients admitted to an ICU showed that the positive predictive value of the C-reactive protein above 50 mg/L and over 150 mg/L for bacterial superinfections, that were microbiologically confirmed, was 61% and 84%, respectively, and 93% for a procalcitonin over 1.00 µg/L [29,30]. Further research is warranted to evaluate the use of these markers for prescribing antibiotics for COVID-19 pneumonia.

It can be difficult to differentiate between the progression of COVID-19 pneumonia and a bacterial superinfection; therefore, the tendency to start an empirical antibiotic treatment can be influenced by previous experiences from influenza pandemics when the co-infection rates were between 11 and 35% [31]. Current guidelines recommend against the use of empirical antibiotics for patients with non-severe COVID-19, unless bacterial infections are laboratory confirmed or are clinically suspected, while for patients with severe disease, empiric antimicrobial therapy can be administered based on the clinical diagnosis, local epidemiology and susceptibility data [22,32].

Our study has the following limitations: the study evaluated only during a 3-month period, which limits the possibility of generalizing the results to the current conditions of the pandemic; not all patients had the same number of microbiologic samples collected (they were collected from patients with clinical findings that were suggestive of bacterial infection) and we only reported the positive results; procalcitonin levels were not evaluated for all patients. Another limitation of our study may be represented by the fact that the treating physicians knew that their patients were included in a clinical study, which could have influenced their decision when prescribing antibiotics (the Hawthorne effect).

## 4. Materials and Methods

### 4.1. Patient Recruitment and Data Collection

**Figure 3:**
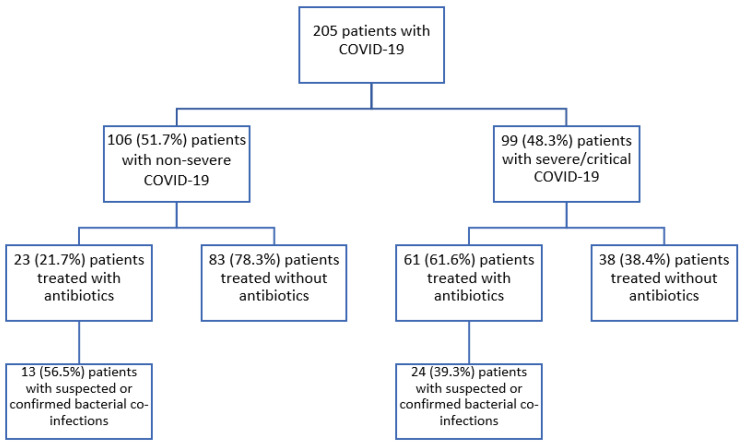
Flow diagram of COVD-19 patients who were treated with or without antibiotics. Suspected or confirmed bacterial co-infections as documented during hospitalization (increased white blood cell count, increased procalcitonin level, bacterial pneumonia aspect on CT scan or X-ray, positive cultures).

In order to estimate the excess of antibiotic prescriptions for patients with COVID-19, and to identify the factors that were correlated with unjustified antibiotic usage, we conducted an observational (cohort) prospective study, in which we enrolled 205 patients with COVID-19 consecutively hospitalized on an infectious disease clinical ward at the National Institute for Infectious Diseases “Prof. Dr. Matei Bals”, from November 2021 to January 2022. All patients signed an informed consent to be enrolled in the study (Figure 3).

Inclusion criteria for this study were as follows: patients over 18 years old, confirmed SARS-CoV-2 infection through nucleic acid detection (RT-PCR) or antigen tests, which required hospitalization. The exclusion criteria were as follows: patients requiring intensive care on admission, patients with end-stage renal disease undergoing hemodialysis or peritoneal dialysis.

To evaluate the prevalence of bacterial co-infection, all positive microbiology results and suspected or confirmed bacterial co-infections (increased white blood cell count, increased procalcitonin level, bacterial pneumonia aspect on CT scan or X-ray, positive cultures), as documented by the treating doctor, were recorded. Depending on the decision to administer antibiotic treatment, the patients were grouped into two categories: patients who received antibiotics and those who did not receive antibiotics, justified or not. Therefore, the dependent variable is the prescription of antibiotics (yes, no) and the independent variables are age, sex, disease severity, fever, C-reactive protein, procalcitonin and leukocyte count.

The data was anonymously collected until the patients were released from the hospital or they died. Patients were grouped in four categories according to the World Health Organization (WHO): with mild disease (without pneumonia or hypoxia), moderate disease (with pneumonia, without hypoxia), severe disease (with pneumonia and hypoxia) and, respectively, critical disease (with acute respiratory distress syndrome, sepsis or septic shock) [33]. For this study, we obtained the approval of the ethics committee of the National Institute for Infectious Diseases “Prof. Dr. Matei Bals”.

### 4.2. Study Endpoints

Our aim was to estimate the excess of antibiotic prescriptions for patients with COVID-19 and to identify the categories of patients that were more frequently associated with unjustified antibiotic usage.

In our study, we defined “adequate treatment” as the antibiotic treatment justified by whether bacterial isolation or at least one clinical or paraclinical findings which indicate bacterial co-infection (leukocytosis, elevated procalcitonin level, CT aspect of bacterial pneumonia) and we defined “inadequate treatment” as the cases in which none of these criteria were met.

### 4.3. Statistical Analysis

In order to compare the two groups of the study (the patients who received antibiotics and those who did not), we used the Mann-Whitney test for continuous variables (age, oxygen flow, number of leukocytes, procalcitonin, etc.) and the Chi-square test or Fisher‘s exact test for categorial variables. All tests were two-tailed and *p*-values of <0.05 were considered statistically significant. Statistics were performed using SPSS Statistics 26. We referred to the STORBE checklist for the reporting of cohort studies. Data are presented as median [IQR] or number (%), as appropriate.

## 5. Conclusions

The ratio of patients treated with antibiotics and those who had clinical or paraclinical findings suggestive of a bacterial infection is higher than desired. Administering antibiotics does not prevent an unfavorable progression of viral pneumonia and does not decrease mortality. Unfortunately, in our country, COVID-19 in-patients received more frequent “watch” group antibiotics. Frequent usage of antibiotics, as well as changes of infection control practices, occurring as a result of the COVID-19 pandemic, contribute to the increase of the cases of infections with antibiotic resistant bacteria. With the emergence of new SARS-CoV-2 strains, it is essential to optimize COVID-19 patient management, along with decreasing the excess prescribing of antibiotics, in the hope that it will limit the increase of antimicrobial resistance. Further research is warranted to better identify the patients that present bacterial co-infection and to initiate antibiotic therapy.

## Figures and Tables

**Table 1:** Baseline characteristics of hospitalized COVID-19 patients treated with and without antibiotics.

*n* = 205	Patients Treated with Antibiotics (*n* = 84)	Patients Treated without Antibiotics (*n* = 121)	*p*-Value
Age, years	70 [54–76]	65 [53–71]	0.023
Male	42 (50.0%)	55 (45.5%)	0.521
Charlson Comorbidity Index	4 [2–5]	2 [1–4]	0.007
Severe/critical COVID-19	61 (72.6%)	38 (31.4%)	<0.001
Clinical features suggestive of bacterial infection	32 (38.1%)	0 (0.0%)	<0.001
Presence of consolidation on CT scan (*n* = 203)	16 (19.3%)	3 (2.5%)	<0.001
Procalcitonin Cut-off: 0.05 (ng/mL) (*n* = 124)	0.05 [0.05–0.16] 31 (48.4%)	0.05 [0.05–0.05] 13 (21.7%)	<0.001 0.002
WBC Cut-off: 9.6 (109/L)	6.2 [4.6–8.6] 16 (19.0%)	5.1 [4.4–6.7] 8 (6.6%)	0.015 0.006
CRP Cut-off: 3.0 (mg/L) (*n* = 185)	69.9 [22.7–119.5]	18.1 [6.1–49.5]	<0.001
Ferritin (ng/mL) Cut-off: 322 (*n* = 132)	536.0 [201.5–936.6]	197.9 [69.8–509.6]	<0.001
IL-6 (pg/mL) Cut-off: 17 (*n* = 135)	127.6 [58.1–463.4]	40.7 [16.7–122.6]	<0.001
Remdesivir	76 (90.5%)	98 (80.9%)	0.06
Glucocorticoids	69 (82.1%)	57 (47.1%)	<0.001
Tocilizumab	11 (13.1%)	4 (3.3%)	0.008
Anakinra	13 (15.5%)	5 (4.1%)	0.005
Baricitinib	10 (11.9%)	7 (5.8%)	0.118
Casirivimab and imdevimab	3 (3.6%)	24 (19.8%)	0.001

WBC = white blood cells, IL-6 = interleukin-6, CRP = C-reactive protein. Data are presented as median [IQR] or number (%), as appropriate.

**Table 2:** Predictors for suspected or confirmed bacterial co-infections in patients diagnosed with COVID-19.

	All COVID-19 Patients (*n* = 205)	Patients with Non-Severe COVID-19 (*n* = 106)	Patients with Severe COVID-19 (*n* = 99)
Symptoms suggestive for a bacterial infection	32 (15.6%)	13 (12.3%)	19 (19.2%)
Pulmonary consolidation on the CT scan (*n* = 203)	19 (9.3%)	4 (3.8%)	15 (15.3%)
White blood cell count Cut-off: >9.6 (10^9^/L)	24 (11.7%)	5 (4.7%)	19 (19.2%)
Procalcitonin (*n* = 124) Cut-off: >0.05 (ng/mL)	44 (35.5%)	15 (24.6%)	29 (46.0%)
C-reactive protein (*n* = 185) Cut-off: >3.0 (mg/L)	169 (91.3%)	82 (85.4%)	89 (97.7%)
Microbiology-proven infections	12 (5.85%)	4 (3.8%)	8 (8.1%)

Procalcitonin was collected within 48 h of admission.

## Data Availability

Not applicable.
